# Antileishmanial Effects of Synthetic *Eh*PIb Analogs Derived from the Entamoeba histolytica Lipopeptidephosphoglycan

**DOI:** 10.1128/AAC.00161-20

**Published:** 2020-06-23

**Authors:** Helena Fehling, Siew Ling Choy, Frederic Ting, Dirk Landschulze, Hannah Bernin, Sarah Corinna Lender, Melina Mühlenpfordt, Eugenia Bifeld, Julia Eick, Claudia Marggraff, Nadine Kottmayr, Marie Groneberg, Stefan Hoenow, Julie Sellau, Joachim Clos, Chris Meier, Hannelore Lotter

**Affiliations:** a Department of Molecular Parasitology and Immunology, Bernhard Nocht Institute for Tropical Medicine, Hamburg, Germany; b Department of Chemistry, University of Hamburg, Hamburg, Germany; c Leishmaniasis Group, Bernhard Nocht Institute for Tropical Medicine, Hamburg, Germany

**Keywords:** *Leishmania*, immunostimulation

## Abstract

With an estimated number of new cases annually of approximately 1.4 million, leishmaniasis belongs to the most important parasitic diseases in the world. Nevertheless, existing drugs against leishmaniasis in general have several drawbacks that urgently necessitate new drug development. A glycolipid molecule of the intestinal protozoan parasite Entamoeba histolytica and its synthetic analogs previously showed considerable immunotherapeutic effects against Leishmania major infection. Here, we designed and synthesized a series of new immunostimulatory compounds derived from the phosphatidylinositol b anchor of *Entamoeba histolytica* (*Eh*PIb) subunit of the native compound and investigated their antileishmanial activity *in vitro* and *in vivo* in a murine model of cutaneous leishmaniasis. The new synthetic *Eh*PIb analogs showed almost no toxicity *in vitro*. Treatment with the analogs significantly decreased the parasite load in murine and human macrophages *in vitro*. In addition, topical application of the *Eh*PIb analog Eh-1 significantly reduced cutaneous lesions in the murine model, correlating with an increase in the production of selected Th1 cytokines. In addition, we could show in *in vitro* experiments that treatment with Eh-1 led to a decrease in mRNA expression of arginase-1 (Arg1) and interleukin 4 (IL-4), which are required by the parasites to circumvent their elimination by the immune response. The use of the host-targeting synthetic *Eh*PIb compounds, either alone or in combination therapy with antiparasitic drugs, shows promise for treating cutaneous leishmaniasis and therefore might improve the current unsatisfactory status of chemotherapy against this infectious disease.

## Abstract (precis)

With an estimated number of new cases annually of approximately 1.4 million, leishmaniasis belongs to the most important parasitic diseases in the world. Nevertheless, existing drugs against leishmaniasis in general have several drawbacks that urgently necessitate new drug development. A glycolipid molecule of the intestinal protozoan parasite Entamoeba histolytica and its synthetic analogs previously showed considerable immunotherapeutic effects against Leishmania major infection.

## INTRODUCTION

Trypanosomatid species of the genus Leishmania cause leishmaniasis, an increasingly prevalent and complex group of poverty-related, neglected tropical diseases that affect over 12 million people worldwide and are endemic in at least 98 tropical and subtropical countries (1). Depending on the infecting species and host immune status, clinical symptoms vary from cutaneous and mucocutaneous forms to the fatal visceral leishmaniasis. Cutaneous leishmaniasis (CL) is the most common form of the disease, causing mostly self-healing ulcers that lead to disfiguring scars, often causing stigmatization (2).

There is no effective vaccine against leishmaniasis, and there are numerous drawbacks to the currently available chemotherapeutics, including the risk of severe side effects, the long treatment duration, emergence of parasite resistance, high costs, and narrow therapeutic windows. Chemotherapeutics include pentavalent antimonials (Pentostam [sodium stibogluconate] and Glucantime [meglumine antimoniate]), the antifungal polyene antibiotic amphotericin B deoxycholate, and its liposomal formulation (AmBisome), which are used in combination or as monotherapies (3–5).

An efficient cure for leishmaniasis might also be achieved by stimulating the host immune response, which is attenuated by the parasite as a survival strategy. By colonizing professional antigen-presenting cells (APCs) such as macrophages, the parasites suppress a protective Th1-type immune response and nitric oxide (NO) production, while promoting a nonprotective Th2-type immune response, e.g., by downregulating the activation of mitogen-activated protein kinases (MAPKs) and induction of arginase synthesis (6, 7). Therefore, activation or reactivation of infected APCs using immunostimulatory compounds represents a promising new therapeutic strategy.

Only a few immunomodulatory drugs have been studied for the treatment of leishmaniasis (8–13). For example, an increased cure rate of CL was observed by treatment with Toll-like receptor 7 (TLR7) agonist imiquimod in human clinical trials and its synthetic analog EAPB0503 in human *in vitro* studies (14, 15). Preclinical studies with CpG D35, an oligodeoxynucleotide containing CpG motifs (CpG ODN), reduced the severity of *Leishmania* infection by TLR9 engagement (16, 17).

We recently reported on the immunostimulatory activity of a lipopeptide phosphoglycan (LPPG) isolated from the membrane of the protozoan Entamoeba histolytica (*Eh*LPPG). This glycolipid activates macrophages by Myd88-dependent TLR or scavenger receptor ligation, resulting in increased interleukin 12 (IL-12) production. Simultaneously, CD1d-mediated presentation of *Eh*LPPG by antigen-presenting cells (APCs) induces gamma interferon (IFN-γ) production by natural killer T (NKT) cells (18, 19). A series of previously synthesized immunostimulatory compounds derived from the phosphatidylinositol (GPI) anchor of the native compound *Eh*LPPG, *Eh*Ia and *Eh*PIb, were found to exhibit antileishmanial activity *in vitro* and *in vivo* in a murine model of CL by inducing synthesis of proinflammatory cytokines (20).

Here, we present a novel set of synthetic analogs based on the *Eh*PIb anchor of *Eh*LPPG that differ in the configuration of the glycerol analogue and the inositol ring, and we assessed their activity against Leishmania major *in vitro* and *in vivo*.

## RESULTS

### Synthesis and chemical structure of the *Eh*PIb analogs.

**FIG 1:**
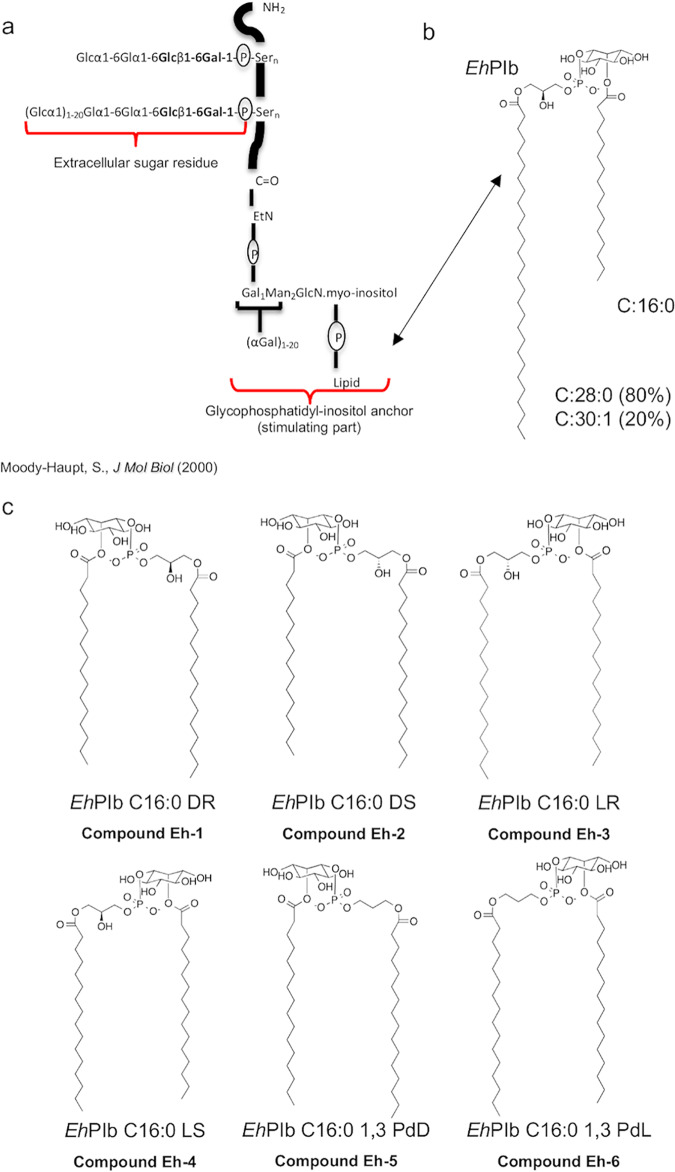
Origin and chemical structure of the synthetic *Eh*PIb analogs. Structure of the native *Eh*LPPG (21) isolated from the membrane of E. histolytica trophozoites (a), which naturally consist of two GPI anchors. The *Eh*PIb anchor (b) served as a template for the design of the synthetic analogs *Eh*PIb C_16:0_ DR (compound Eh-1), *Eh*PIb C_16:0_ DS (compound Eh-2), *Eh*PIb C_16:0_ LR (compound Eh-3), *Eh*PIb C_16:0_ LS (compound Eh-4), *Eh*PIb C_16:0_ 1,3-PdD (compound Eh-5), and *Eh*PIb C_16:0_ 1,3-PdL (compound Eh-6) (c). Abbreviations: EtN, ethanolamine; GLA, alpha-galactosidase; GAL, galactose; GlcN, glucosamine; Man, mannose.

All six synthetic analogs were derived from the *Eh*PIb anchor of *Eh*LPPG (18, 21), which was isolated from the membrane of E. histolytica trophozoites (Fig. 1a). *Eh*PIb has a phosphatidylinositol scaffold, which has a long-chain fatty acid of either 30 monounsaturated carbons (C_30:1_) or 28 saturated carbons (C_28:0_), as well as an additional short-chain fatty acid (C_16:0_) at the stereospecifically numbered 2-position (sn-2 carbon atom) of the *myo*-inositol moiety (20) (Fig. 1b). In the synthetic derivatives, the long-chain fatty acid is exchanged by a C_16_ short-chain fatty acid (Fig. 1c).

The inositol moiety of the derivatives Eh-1 and Eh-2 have a d-configuration, whereas the inositol moieties of the derivatives Eh-3 and Eh-4 are l-configurated. While the chiral C-2 atoms in the glycerol moieties of Eh-1 and Eh-3 are (*R*)-configurated, the corresponding configuration in Eh-2 and Eh-4 is (*S*). The derivatives Eh-5 and Eh-6 lack the OH group at the C-2 atom in the glycerol moiety, which results in the removal of the chiral center.

The detailed reaction scheme for the synthesis of these analogs, as well as the reaction conditions and analytical data, are provided in the supplemental material.

### Cytotoxicity of synthetic *Eh*PIb analogs.

**FIG 2:**
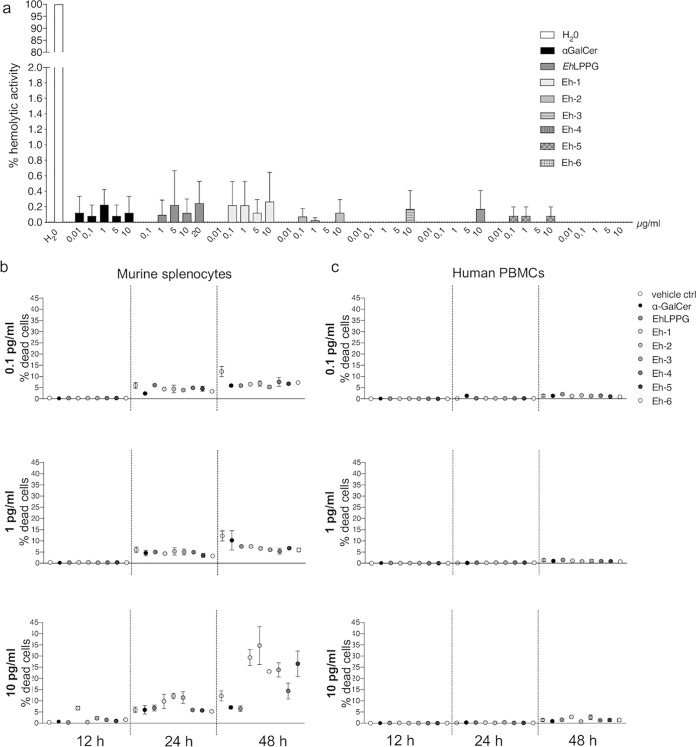
Toxicity of α-GalCer, *Eh*LPPG, or the synthetic *Eh*PIb analogs *in vitro*. Hemolytic activity of α-GalCer, *Eh*LPPG, or the synthetic *Eh*PIb analogs (0.01/0.01/0.1/1/5/10 μg/ml) against human red blood cells after 1 h of incubation (a). Percentage of dead murine splenocytes (b) and human peripheral blood mononuclear cells (PBMCs) (c) after 12, 24, and 48 h of incubation with the synthetic analogs (0.1/1/10 μg/ml), as determined by fluorescence-activated cell sorting (FACS). Results are expressed as the mean ± standard deviation (SD) of 2 or 3 independent experiments (*n* = 2 or 3/experiment).

To compare the cytotoxic properties of synthetic *Eh*PIb analogs with those of the native *Eh*LPPG molecule, their hemolytic activity against human erythrocytes and their impact on the viability of murine lymphocytes (splenocytes) and human peripheral blood mononuclear cells (PBMCs) was measured (Fig. 2).

Erythrocytes were incubated with *Eh*PIb analogs (0.01 μg/ml, 0.1 μg/ml, 1 μg/ml, and 10 μg/ml), *Eh*LPPG, and the NKT cell stimulator α-galactosylceramide (α-GalCer) (22), and the hemolytic activity was measured photometrically in the supernatant. All molecules showed very low hemolytic activity (<0.5%) (Fig. 2a). Next, murine splenocytes or human PBMCs were incubated with the compounds, and the cytotoxicity was determined by flow cytometry based on live/dead staining (Fig. 2b and c). Various levels of cytotoxicity were observed, depending on the molecule concentration and incubation time (Fig. 2b). Eh-2 exhibited the highest toxicity in murine splenocytes (34.7% dead cells at 10 μg/ml and 48 h). In contrast, most of the compounds induced minimal toxicity of human PBMCs (2 to 4% dead cells at 48 h; Fig. 2c). In conclusion, the synthetic analogs have no significant cytotoxicity toward host cells.

### *In vitro* activities of synthetic *Eh*PIb analogs against L. major infection.

**FIG 3:**
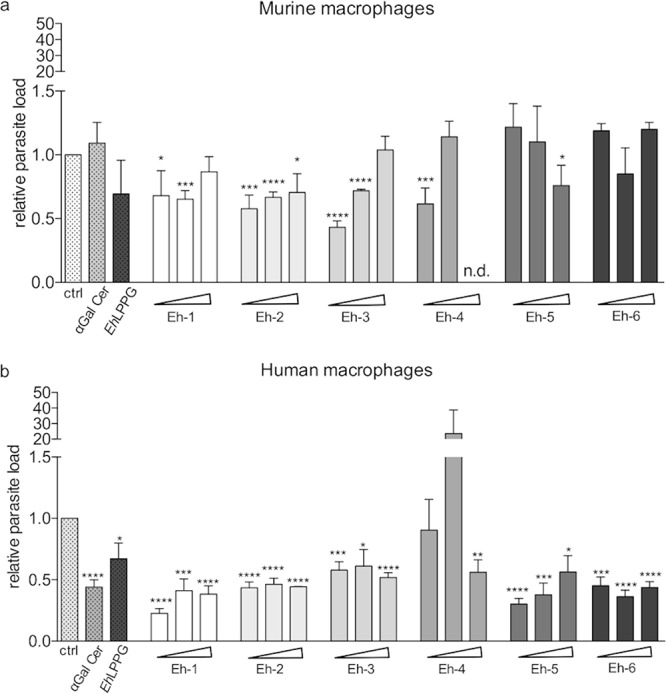
Activity of α-GalCer, *Eh*LPPG, and the synthetic *Eh*PIb analogs against L. major *in vitro*. Murine bone marrow-derived macrophages (BMDMs) and human THP1 cells were infected with stationary-phase promastigotes (at a multiplicity of infection [MOI] of 8 parasites per macrophage) and then treated with α-GalCer (4 μg/ml), *Eh*LPPG (8 μg/ml), or the synthetic *Eh*PI analogs (0.1/1.0/10.0 μg/ml). Genomic DNA was extracted from infected and treated macrophages and used in a TaqMan probe quantitative PCR (qPCR). To determine the relative parasite load in murine (a) and human (b) macrophages, the 2^−ΔΔ^*^CT^* method was used and normalized to the infected control without treatment. Data are expressed as the mean ± standard error of the mean (SEM) of three independent experiments (*n* = 2/experiment). *, *P* < 0.05; **, *P* < 0.01; ***, *P* < 0.001; ****, *P* < 0.0001 (unpaired Student’s *t* test).

We previously reported that *Eh*LPPG and the first set of synthetic *Eh*PI analogs reduced parasite loads in L. major-infected macrophages (20). Here, we tested the antileishmanial activity of the newly synthesized compounds Eh-1, Eh-2, Eh-3, Eh-4, Eh-5, and Eh-6 against L. major-infected bone marrow-derived macrophages (BMDMs) and human THP1 cells. Relative parasite loads were determined after 48 h by real-time PCR using duplex TaqMan PCR (23). The synthetic analog Eh-2 caused a significant parasite load reduction at all tested concentrations (0.1 to 10 μg/ml, *P* < 0.015) in murine and human cells. Eh-1 only reduced the parasite load at 0.1 to 1 μg/ml (*P* ≤ 0.035), while Eh-5 reduced the parasite loads only at 10 μg/ml and Eh-4 only at 0.1 μg/ml. Eh-3 significantly reduced the parasite load at 0.1 to 1 μg/ml. Treatment with α-GalCer, *Eh*LPPG, or Eh-6 had no effect at any concentration on the parasite load in murine macrophages (Figure 3a).

In contrast, α-GalCer, *Eh*LPPG, and five of the synthetic *Eh*PIb analogs significantly reduced the parasite load in human THP1 macrophages at all concentrations tested (0.1 to 10 μg/ml, *P* < 0.015; Fig. 3b). We therefore find considerable antileishmanial activity for the majority of the synthetic molecules.

### Activity of compound Eh-1 against L. major infection *in vivo*.

**FIG 4:**
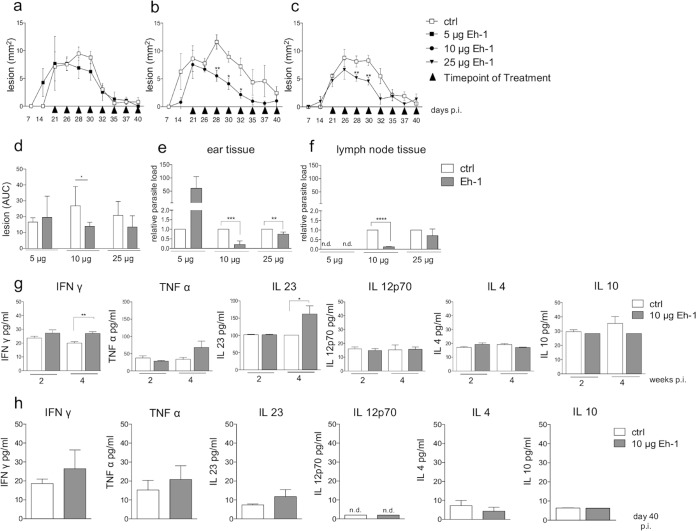
Activity of compound Eh-1 against L. major *in vivo*. Course of L. major-induced ear lesions in female C57BL/6 mice (*n* = 5 or 6) topically treated with 5 μg (a), 10 μg (b), or 25 μg (c) of Eh-1 at indicated points of time postinfection (p.i.). The disease burden was determined based on the area under the curve (AUC) (d). Parasite load in the ear tissue (e) and in the draining lymph nodes (f) of L. major-infected C57BL/6 mice was determined by qPCR. Cytokine profiles of blood serum were analyzed two and 4 weeks p.i. (g), as well as cytokine profiles of ear tissue lysates from the infected animals on day 40 p.i. (h), as determined in a cytometric bead assay (LEGENDplex; BioLegend). Results are expressed as the mean ± SEM. *, *P* < 0.05; **, *P* < 0.01; ***, *P* < 0.001; ****, *P* < 0.0001 (unpaired Student’s *t* test).

We next examined the therapeutic effect of Eh-1 in a murine model of CL. L. major promastigotes were injected intradermally into the outside auricle of C57BL/6 mice. After the onset of swelling (14 to 20 days postinfection [p.i.]), Eh-1 was applied topically three times a week at three dose levels (5 μg, 10 μg, or 25 μg) (Fig. 4a to c). Compared with the vehicle control (dimethyl sulfoxide [DMSO]), treatment with 5 μg Eh-1 slightly reduced the ear swelling between day 28 and day 32, but not after that time (Fig. 4a). In contrast, treatment with 10 μg of the compound significantly reduced the lesion size up to day 32 (Fig. 4b). Treatment with 25 μg Eh-1 also significantly reduced the lesion size between day 28 and day 30 (*P* ≤ 0.01). The final healing process paralleled that of the vehicle-treated mice (Fig. 4c).

The disease burden was determined based on the area under the curve (AUC) (Fig. 4d). In comparison with the vehicle control, there was a significant reduction in the disease burden after treatment with 10 μg/ml Eh-1. Quantification of the parasite load using quantitative PCR (qPCR) at the end of the experiment (day 40) revealed a significant reduction in parasite numbers in the skin (*P* < 0.001) and the lymph nodes (*P* < 0.001) in the 10-μg treatment group and in the skin (*P* < 0.01) in the 25-μg treatment group (Fig. 4e and f). Analysis of the cytokine profile in the serum of mice treated with 10 μg Eh-1 revealed a significant increase of the proinflammatory cytokines IFN-γ (*P* ≤ 0.01) and IL-23 (*P* ≤ 0.1) after 2 weeks of treatment (Fig. 4g). The cytokine profiles of the ear tissues showed no significant cytokine induction but a slight increase in IFN-γ, tumor necrosis factor alpha (TNF-α), and IL023 (Fig. 4h). In summary, these data show a considerable therapeutic effect of topical treatment with a synthetic *Eh*PIb analog, correlating with increased proinflammatory cytokines in the serum.

### Localization of iNOS- and arginase-expressing immune cells in L. major-infected C57BL/6 mice.

**FIG 5:**
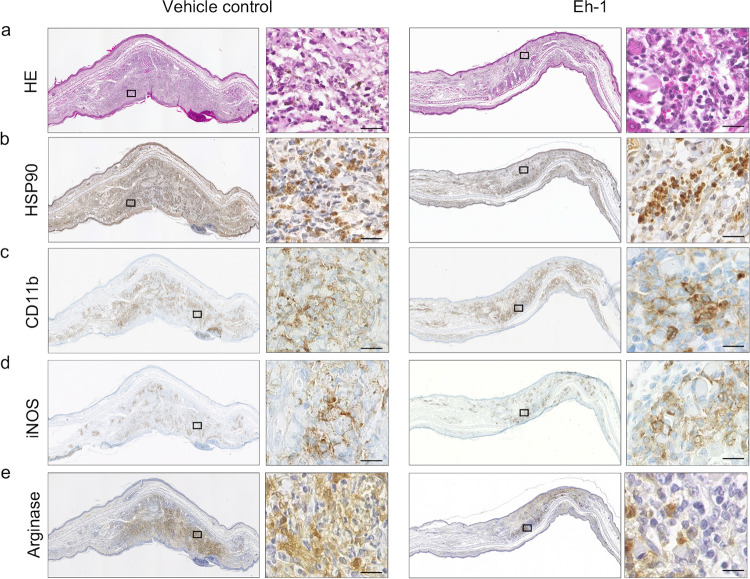
Immunohistological characterization of cellular infiltrate, iNOS, and Arg1 expression in L. major-infected C57BL/6 mice post topical treatment with compound Eh-1. Sequential slices of paraffin-embedded ear tissue sections from L. major-infected C57BL/6-mice, topically treated with 10 μg Eh-1 at day 40 p.i. were stained with hematoxylin and eosin (H&E) (a), anti-HSP90 (L. major) (b), CD11b-staining (monocytes and macrophages) (c), iNOS (d), and arginase-1 (Arg1) (e) and compared to the respective vehicle control. An overview is shown in low magnification (×10) and the same ear area is presented in a higher magnification (×60). Bar, 10 μm. Representative staining of 1 out of 3 animals/group analyzed.

**FIG 6:**
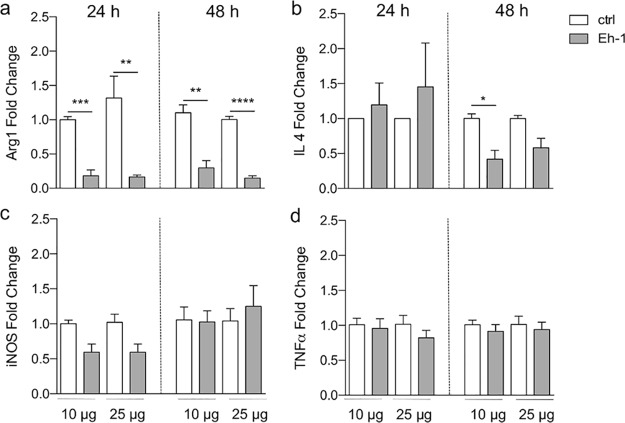
Treatment-induced macrophage changes in mRNA expression. Arg1 (a), IL-4 (b), iNOS (c), and TNF-α (d) mRNA expression fold change (qPCR relative to glyceraldehyde-3-phosphate dehydrogenase [GAPDH] or ribosomal protein S9 [RPS9]) in bone marrow-derived macrophages (BMDMs) infected with stationary-phase L. major promastigotes (at an MOI of 8 parasites per macrophage) and treated 24 h postinfection with compound Eh-1 (10 μg, 25 μg) for 24 and 48 h compared to vehicle control. Results are expressed as the mean ± SEM. *, *P* < 0.05; **, *P* < 0.01; ***, *P* < 0.001; ****, *P* < 0.0001 (unpaired Student’s *t* test) (*n* = 3 to 5).

Immunohistological staining was performed to characterize the impact of Eh-1 on the immune cell infiltrate and the expression of crucial enzymes involved in intracellular control of *Leishmania* parasites, such as inducible nitric oxide synthase (iNOS) and arginase (Fig. 5a to e). Combined hematoxylin and eosin (H&E) and anti-HSP90 staining showed reduced ear swelling in Eh-1-treated mice compared with DMSO-treated mice, but a comparable amount of infiltrating immune cells and *Leishmania* parasites (Fig. 5a and b). Tissue from Eh-1-treated mice showed similar amounts of CD11b^+^ monocytes and macrophages in treated and nontreated mice (Fig. 5c). We observed a minor treatment-dependent increase in the expression of iNOS in CD11b^+^ cells of treated mice (Fig. 5d). Conversely, the expression of the nonprotective effector molecule arginase was reduced in treated mice (Fig. 5e). This treatment-induced reduction of arginase was also observed, as well as a minor decrease of iNOS after 48 h of treatment *in vitro*, using quantitative reverse transcription-PCR (RT-PCR) (Fig. 6a and c). To further characterize the treatment-induced immunostimulatory effects, we determined the mRNA levels of the infection-relevant cytokines IL-4 and TNF-α (Fig. 6b and c). However, Eh-1 treatment had no effect on TNF-α levels, but resulted in a significant reduction 48 h posttreatment (10 μg/ml). Taken together, treatment with Eh-1 reduced the expression of arginase by infiltrating myeloid cells *in vitro* and *in vivo*.

## DISCUSSION

Immunotherapy is a promising strategy for the treatment of infectious diseases. Host targets for immunotherapy would ideally interfere with pathogen invasion, survival, and/or replication.

We previously demonstrated that the immunostimulatory glycolipid *Eh*LPPG and its synthetic analogs induce protective cytokine responses in immune cells as well as anti-parasitic effects in experimental *in vitro* and *in vivo* models of L. major infection (18, 20). *Eh*LPPG elicits its immunostimulatory effects via two mechanisms. First, TLR2/TLR6 engagement and Myd88 signaling induce the production of proinflammatory cytokines such as IL-12. Second, uptake of the glycolipid leads to processing and loading onto CD1d molecules in the late endosomes of APCs. Subsequent presentation to invariant NKT cells, which express a semi-invariant α T-cell receptor (αTCR) paired with a limited repertoire of αTCR chains, elicits a strong immune response that leads to functions that are crucial for the control of several infectious diseases, including leishmaniasis (18, 24–26). This unique, dual mode of immune activation makes *Eh*LPPG highly attractive for further development as an immunotherapeutic drug.

The synthetic analogs of *Eh*PIb all consist of two short C_16:0_ fatty acids with different configurations of the phosphatidylinositol and the glycerol moiety (Fig. 1c) and are therefore optimally suited for CD1d loading on APCs (27). Native or synthetic analogs of *Eh*LPPG comprising fatty acids with attached hydrophobic and hydrophilic structures require good solubility, and their ability to form micelles may cause insertion into and rupture of the lipid bilayer of eukaryotic cell membranes, resulting in cytotoxicity (28). We therefore examined the hemolytic activity and cytotoxicity of the synthetic compounds *in vitro.* Consistent with earlier results (20), α-GalCer and *Eh*LPPG were weakly hemolytic, while the synthetic analogs showed minimal hemolytic activity and negligible cytotoxicity against human peripheral blood lymphocytes or murine splenocytes (Fig. 2).

Due to their structural similarities with *Eh*LPPG, we expected the synthetic analogs to activate APCs such as macrophages and to induce a protective immune response. Since macrophages are the main target cells for *Leishmania*, as well as the main effector cells for parasite clearance, we investigated the antileishmanial activity of the synthetic analogs in L. major-infected murine and human macrophages. Eh-1, Eh-2, and Eh-3 were the most potent compounds in murine macrophages, while all synthetic analogs except for compound Eh-4 showed strong activity in human macrophages. These variations might be related to the use of different cells in our study, primary murine cells versus immortalized human cell lines, as our findings are in accordance with previous observations that showed stronger antileishmanial effects using human macrophages or immortalized cell lines (29, 30). Even species-specific characteristics of TLR expression and divergences in the metabolic response could influence the efficacy of the synthetic analogs and therefore lead to a stronger activation of human macrophages (31, 32). The antileishmanial effect of most synthetic analogs was not dose dependent, a phenomenon observed previously in both murine and human cells (20). We suspect that the compounds form micelles at higher concentrations. We plan to test incorporation of the compounds into suitable nanocarriers to overcome this problem. However, compared with the previously described set of synthetic analogs (20), the new compounds show increased antileishmanial activity, suggesting that the receptor affinity of the molecules is contingent on their molecular structure. In addition, topical treatment with one selected synthetic analog (compound Eh-1) in a mouse model of experimental CL significantly reduced ear swelling (Fig. 4b, c), the disease burden (Fig. 4d), and the parasite loads in the ear and in lymph node tissues (Fig. 4e, f). C57BL/6 mice are considered the most suitable model for clinical evaluation of drug candidates for CL due to the relative resistance of the strain against L. major, resulting in clinical symptoms close to those in humans (33). Our data indicate that Eh-1 stimulates the immune system of C57BL/6 mice to eliminate *Leishmania* parasites by activating macrophages and possibly other immune cells, leading to a protective Th1 immune response. Interestingly, and despite the topical route of administration, we found increased serum levels of cytokines involved in host protection against CL (TNF-α, IFN-γ, and IL-23) following treatment with Eh-1 (34–36). This may be due to a strong, local activation of skin NKT cells, which can promote or inhibit adaptive immune responses (37). However, a suppressive role for NKT cells in allergic reactions has been described (38, 39). The recruitment and activation of resident and peripheral APCs, respectively, may also amplify the local immune response (34, 40).

Resistance to experimental leishmaniasis correlates with a Th1-type immune response and the production of proinflammatory cytokines, resulting in parasite killing, whereas susceptibility is associated with a Th2-type immune response that allows parasite replication and persistence (41). In this study, immunohistological staining of L. major-infected ear tissues showed that CD11b^+^ cells expressed iNOS and arginase, suggesting that M1 as well as M2 macrophages were present at the site of infection. Since arginase-expressing cells were detected in the control animals, we conclude that the numbers of M2 macrophages were higher in these animals, possibly explaining why *Leishmania* infection was not controlled as efficiently as in Eh-1-treated animals. Treatment-induced arginase and IL-4 reduction was also observed *in vitro*. While M1 macrophages induce iNOS, which converts the substrate l-arginine to NO, resulting in parasite elimination, M2 macrophages specialize in the production of arginase, which hydrolyses l-arginine to ornithine, a basic building block of polyamine biosynthesis, and are therefore crucial for parasite survival (42). We assume that treatment with the synthetic analogs activates and polarizes macrophages via the classical pathway, which is mediated by cytokines such as INF-γ and ultimately results in antileishmanial activity.

### Conclusion.

We show that synthetic analogs derived from the immunostimulatory glycolipid *Eh*LPPG constitute leads for a potential treatment of CL by enhancing the host immune response. Treatment with the synthetic analogs lowered the L. major parasite load *in vitro* and *in vivo*, reduced the clinical symptoms of infected mice, and induced a protective Th1 immune response. Nevertheless, further studies are needed to examine the exact mechanism of action of these compounds and to explore their potential combinatorial effects with antiparasitic drugs.

## MATERIALS AND METHODS

### Synthesis of *Eh*PIb analogs.

The *Eh*PIb analogs were synthesized in a convergent synthetic route starting from *myo*-inositol and d-mannitol using phosphor diamidite 13 as coupling agent (see supplemental material).

The precise nomenclature of the *Eh*PIb analogs is presented in Fig. 1c. In order to simplify the nomenclature, we have named all *Eh*PIb analogs compounds Eh-1 to Eh-6.

### Ethics.

All animal experiments were carried out in accordance with the guidelines adhering to the NHI institutional and animal research for the care and use of laboratory animals (ARRIVE) and approved by the review board of the State of Hamburg, Germany (acquisition no. 46/13 and 133/13). The animals were bred and kept under pathogen-free conditions at the Bernhard Nocht Institute for Tropical Medicine (BNITM), Hamburg, Germany.

All experiments with human samples were approved in accordance with relevant guidelines and regulations. The BNITM and the medical council of Hamburg authorized the experimental protocols, and all donors provided informed consent.

### Parasite culture.

L. major (MHOM/SU/73/*5*ASKH) cultures were routinely grown at 25°C in modified medium 199 (Sigma-Aldrich, with Hanks’ salts, 20% heat-inactivated fetal calf serum, 40 nM HEPES [pH 7.4], 0.2% NaHCO_3_, 100 μM adenine, 1.2 μg/ml 6-biopterin, 10 μg/ml heme, 20 μg/ml gentamicin, and 2 mM l-glutamine [pH 7.0]). For all experiments, parasites were allowed to grow to the stationary phase and then counted using a Casy cell counter (Roche).

### Preparation of the synthetic analogs for *in vitro* and *in vivo* experiments.

The compounds Eh-1 to Eh-6 were dissolved in DMSO and stored at −20°C. Before use, the synthetic analogs were sonicated in a 37°C preheated ultrasonic bath (Sonorex Super DK 255; Bandelin) for 10 min. Due to the structural similarities, and, correspondingly, the same way of preparation before use, *Eh*LPPG and α-GalCer (Enzo Life Sciences) were used as reference stimulants. DMSO was used as a vehicle control. For *in vitro* experiments, the compounds were diluted in the respective culture medium, and for *in vivo* experiments, the compounds were diluted in Dulbecco’s phosphate-buffered saline (DPBS).

### Hemolytic activity of synthetic analogs.

To investigate the hemolytic activity of Eh-1 to Eh-6, as well as that of the reference stimulants α-GalCer and *Eh*LPPG, erythrocytes were incubated with different concentrations (ranging from 0.1 to 20 μg/ml) of the compounds as described previously (20). The absorbance of the supernatant was measured at 530 nm in an enzyme-limited immunosorbent assay (ELISA) counter (MRX; Dynex, Magellan Bioscience) with the reference filter set at 630 nm. The percentage of hemolytic activity was determined as follows: [(*A* − *A*_0_)/(*A*_max_ − *A*_0_)] × 100. *A*_0_ represents the background hemolysis obtained by incubation of erythrocytes with PBS, and *A*_max_ represents 100% hemolysis achieved upon incubation of erythrocytes in distilled water.

### Cytotoxicity assay of synthetic analogs in murine and human lymphocytes.

For cytotoxicity testing, murine splenocytes and human PBMCs were isolated as previously described (18, 20). Murine or human lymphocytes (1 × 10^6^) were incubated and stimulated with different concentrations (0.1, 1.0, or 10.0 μg/ml) of compounds Eh-1 to Eh-6 and the reference stimulants α-GalCer and *Eh*LPPG for 12, 24, or 48 h at 37°C and 5% CO_2_. In this experiment, three controls were used, namely, a medium control, a vehicle (DMSO) control, and a control for Zombie UV-positive cells (dead cells). The positive control was generated by exposing 5 × 10^5^ cells to 95°C for 15 min in order to kill the cells. After incubation, cells were stained (Zombie UV fixable viability kit, BioLegend) according to the manufacturer’s instructions. Data acquisition was performed using a BD LSR II flow cytometer.

### *In vitro* infection of murine and human macrophages with L. major.

Murine bone marrow-derived macrophages (BMDMs) of 6- to 10-week-old female BALB/c or C57BL/6J mice were generated by a modified protocol after Racoosin (1989) as described previously by Choy et al. (20) for *in vitro* infection. For human *in vitro* infection, THP1 cells (ATCC TIB-202) were used.

After differentiation, adherent BMDMs (1.5 × 10^6^ cells/well; 24-well plate) or THP1 cells (6 × 10^5^ cells/well; 24-well plate) were infected with L. major promastigotes (multiplicity of infection [MOI], 8 parasites:1 macrophage) as described previously (20, 43). Infected murine or human macrophages were treated with 0.1, 1.0, or 10 μg/ml of compounds Eh-1 to Eh-6, as well as with 4.0 μg/ml α-GalCer and 8.0 μg/ml *Eh*LPPG. After 48 h of treatment, murine or human macrophages were subjected to genomic DNA (gDNA) isolation (QIAmp gDNA kit; Qiagen) according to the manufacturer’s instructions (23, 44).

For total cellular RNA isolation, BMDMs (2 × 10^5^ cells/well) were seeded into 12-well plates (Sarstedt) and further processed as described above. Postinfection, cells were incubated for 24 h at 37°C and 5% CO_2_ and then treated for 24 h and 48 h with 10 μg/ml and 25 μg/ml of compound Eh-1.

### Quantitative RT-PCR.

In order to quantify the relative parasitic burden in murine and human macrophages, probe PCR (Kapa Probe Fast universal qPCR mastermix; Peqlab) was performed on the host/parasite gDNA mix. Parasite burden was calculated using the 2^−ΔΔ^*^CT^* method (45) and normalized to the infected control (set as 1.0).

For detection of arginase-1 (Arg1), iNOS, TNF-α, and IL-4 mRNA, total cellular RNA was isolated using the InviTrap SpinCell RNA minikit (Stratec Molecular), and cDNA was synthesized using Maxima First Strand cDNA synthesis kit (Thermo Scientific). Quantitative RT-PCR was performed using Maxima SYBR green qPCR mastermix (Thermo Scientific). The data were analyzed using the 2^−ΔΔ^*^CT^* method and normalized to the housekeeping gene encoding glyceraldehyde-3-phosphate dehydrogenase (GAPDH) or to the housekeeping gene encoding ribosomal protein S9 (RPS9). Primers used for probe PCR were described previously by Choy et al. (20). The following primers (5′ to 3′) were used for mRNA detection at a final concentration of 0.3 μM: arginase-1: mARG1 for, AACACTCCCCTGACAACCAG and mARG1 rev, CCAGCAGGTAGCTGAAGGTC; miNOS for, TGGTGGTGACAAGCACATTT and miNOS rev, AAGGCCAAACACAGCATACC; mTNFα for, AGTTCCCAAATGGCCTCCCTCTCA and mTNFα rev, GTGGTTTGCTACGACGTGGGCT; mIL 4 for, CCAAGGTGCTTCGCATATTT and mIL 4 rev, ATCGAAAAGCCCGAAAGAGT.

### C57BL/6 mouse model of cutaneous leishmaniasis.

For infection 3 × 10^6^ L. major promastigotes were injected intradermally in 10 μl of PBS into the ear of 8- to 12-week-old female C57BL/6 mice. The course of infection was determined by measuring the ear swelling using photo analysis with Fiji software (ImageJ) three times a week. When ear swelling started to develop, the animals were treated three times a week with the compound Eh-1 at concentrations of 5, 10, and 25 μg in 10 μl of DPBS topically applied to the ear.

After reduction of the ear swelling, the mice were sacrificed, and ear tissue, as well as lymph node tissue, was used for histological staining and gDNA isolation. Blood serum was taken every 2 weeks p.i. The cytokine profile of the serum, as well as that of the tissue lysates, was analyzed using a LEGENDplex assay kit (BioLegend). Lysates of the infected ears were obtained by mincing with zirconia beads (2 mm; Carl Roth GmbH) and incubated on ice with 200-μl protease inhibitor tablets (Sigma-Aldrich). Samples were then mixed in a TissueLyser for 10 min at 50 ms and then centrifuged at 4°C at maximum speed. The supernatant was used for cytokine analysis.

### Immunohistology.

Sequential slices of paraffin-embedded L. major-infected ear tissue were immunohistologically stained with hematoxylin and eosin (H&E). The *Leishmania* parasites were stained using an anti-mouse HSP90-antibody (heat shock protein, 1:600) in combination with a polyclonal anti-mouse IgG-second antibody, linked to horseradish peroxidase (HRP) (1:200; Dako). Arginase was stained with an anti-mouse Arg1-antibody (BD Biosciences) in combination with the M.O.M. immunodetection kit (Vector). The enzyme iNOS was visualized with a polyclonal anti-mouse iNOS-antibody (Abcam), whereas monocytes and macrophages were stained with monoclonal anti-mouse CD11b-antibody (Abcam). The targets were visualized with a Ventana diagnostic systems kit (Roche).

### Statistical analysis.

Percentages of cytokine expression were compared between either untreated and treated murine or human cells or between infected and infected/treated cells by unpaired Student's *t* test. Parasite load and ear lesion sizes were also compared between infected and infected/treated samples and mice by unpaired Student’s *t* test. Differences were considered to be significant if the *P* values were as follows: *, *P* < 0.05; **, *P* < 0.01; ***, *P* < 0.001; ****, *P* < 0.0001. The normal distribution of the data was confirmed using the Shapiro-Wilk test as part of the GraphPad Prism statistic software version 8.0.2.

## Supplementary Material

Supplemental file 1
