# Supplementary material for: Antileishmanial Effects of Synthetic EhPIb Analogs Derived from the Entamoeba histolytica Lipopeptidephosphoglycan
Source: Antimicrob Agents Chemother. 2020 Jun 23;64(7):e00161-20. doi: 10.1128/AAC.00161-20 (PMC7318009; doi:10.1128/AAC.00161-20)
Supplement: Supplemental file 1 [file AAC.00161-20-s0001.pdf]

Supplementary information

Supplementary text

Chemical synthesis of the synthetic analogs

We developed a convergent route for the synthesis of six optically pure *Eh*PIb-type analogs Eh-1 (**1a**), Eh-2 (**1b**), Eh-3 (**1c**), Eh-4 (**1d**), Eh-5 (D-**2**) and Eh-6 (L-**2**)**.** These were prepared from precursors **3** and **4** or **3** and **5** via a phosphoramidite-mediated coupling starting from *myo*-inositol **6**. *Myo*-inositol **6** was selectively transformed into racemic *tetra*-benzylated *myo*-inositol **7**, firstly by blocking positions 1 and 2 with 2,2-DMP (1) and secondly by addition of four benzyl groups to the positions 3,4,5,6 and deprotection of the acetal (2) which gave racemic diol (*rac*)-**7** in a yield of 41% (three steps, Figure S1a).

In order to obtain the two enantiomers of (*rac*)-**7** separately, a chiral resolution of the racemic mixture was carried out by transforming the racemate into a mixture of the diastereomeric esters **8a** and **8b** followed by separation of this diastereomeric mixture via flash column chromatography (Figure S1b).

The diastereomeric esters (*mix*)-**9** were synthesized by using (1*S*)-(-)-camphanic chloride (Table S1, #1). As described by Nkambule *et al.* (3). The diastereomers of the *O*-1 regioisomer were found to be non-separable.Thus, we used the reaction conditions described by Nkambule et al. to migrate the acyl group from *O*-1 to *O*-2.A mixture of the 1-substituted regioisomers **9** and 2-substituted regioisomers **8** were yielded in a ratio of 7:3 in favour of the desired regioisomers **8**. These were then separated using silica gel column chromatography to obtain **8a** and **8b** in yields of 30% and 27%, respectively (Table S1, #2, #3). The ester groups were cleaved in sodium hydroxide solution which resulted diols **D-7** and **L-7** in good yields of 92% and 93% (Table S1, #4, #5) (4). Both enantiomers were selectively allylated using dibutyltin(IV) oxide (5) to afford the allyl ethers **D-10** and **L-10** in good yields (Table S1, #6, #7), which were then acylated with palmitic chloride to give **D-11** and **L-11** (Table S1, #8, #9). Next, the fully protected *myo*-inositols were deallylated using palladium(II)chloride and

the alcohols D-**12** and L-**12** were obtained in moderate yields (Table S1, #10, #11)(6). In the following step the phosphoramidites D-**3** and L-**3** were prepared using benzyl protected phosphordiamidite **13** which gave the *myo*-inositol precursors D-**3** and L-**3** in good yields (Table S1, #12, #13).

The glycerol precursors (*R*)-**4** and (*S*)-**4** were prepared starting from commercially available D-mannitol **14**. D-Mannitol **14** was converted into a dibenzyl protected compound **15** in three steps via benzylidene acetal **16** and fully protected mannitol **17** (Table S2). Starting from tetrol **15** two routes were developed to generate precursors (*S*)-**4** or (*R*)-**4**. In the first method for the synthesis of (*S*)-**4,** tetrol **15** was acylated twice to afford *cis*-diol **18**, followed by sodium periodate mediated oxidative cleavage and reduction of the resulting aldehyde which gave *sn*-1-acylated alcohol (*S*)-**4** (Table S2). The slightly longer synthesis route for (*R*)-**4** consisted of two additional steps to direct the *sn*-3 position for acylation. Tetrol **15** was first silylated to yield silyl ether 19 which was then transformed into two equivalents of alcohol **20** via oxidative cleavage as described above. After acylation the fully protected glycerol **21** was desilylated in mild conditions to yield *sn*-3-acylated (*R*)-isomer (*R*)-**4**.

With both precursors now prepared, phosphoramidites D-**3**, L-**3** and the alcohols (*R*)-**4** and (*S*)-**4** were coupled to the four diastereomers **22a-d** in yields of 51%-85% (Table S3). The fully protected phosphatidylinositols **22a-d** were then deprotected by hydrogenolysis with palladium-black obtaining the PIs **1a-d** in moderate yields (Figure S1, Table S3).

The syntheses of the derivatives D-**2** and L-**2** were carried out as described above using 1,3-propanediol instead of glycerol. 1,3-Propanediol lacks the 2-hydroxy group compared with glycerol and therefore its unsymmetrically substituted derivatives are not chiral. For this reason the synthetic route for D-**2** and L-**2** were significantly shorter compared to **1a-d**. In the first step 1,3-propanediol was monopalmitoylated using palmitoyl chloride to yield compound **5**. The coupling to the amidite precursors D-**3** and L-**3** was done as described above to afford

D-**24** and L-**24**. After cleavage of the benzyl groups by hydrogenolysis as described above, phosphatidylinositols D-**2** and L-**2** were obtained in good yields (Figure S1, Table S3).

Supplementary figures

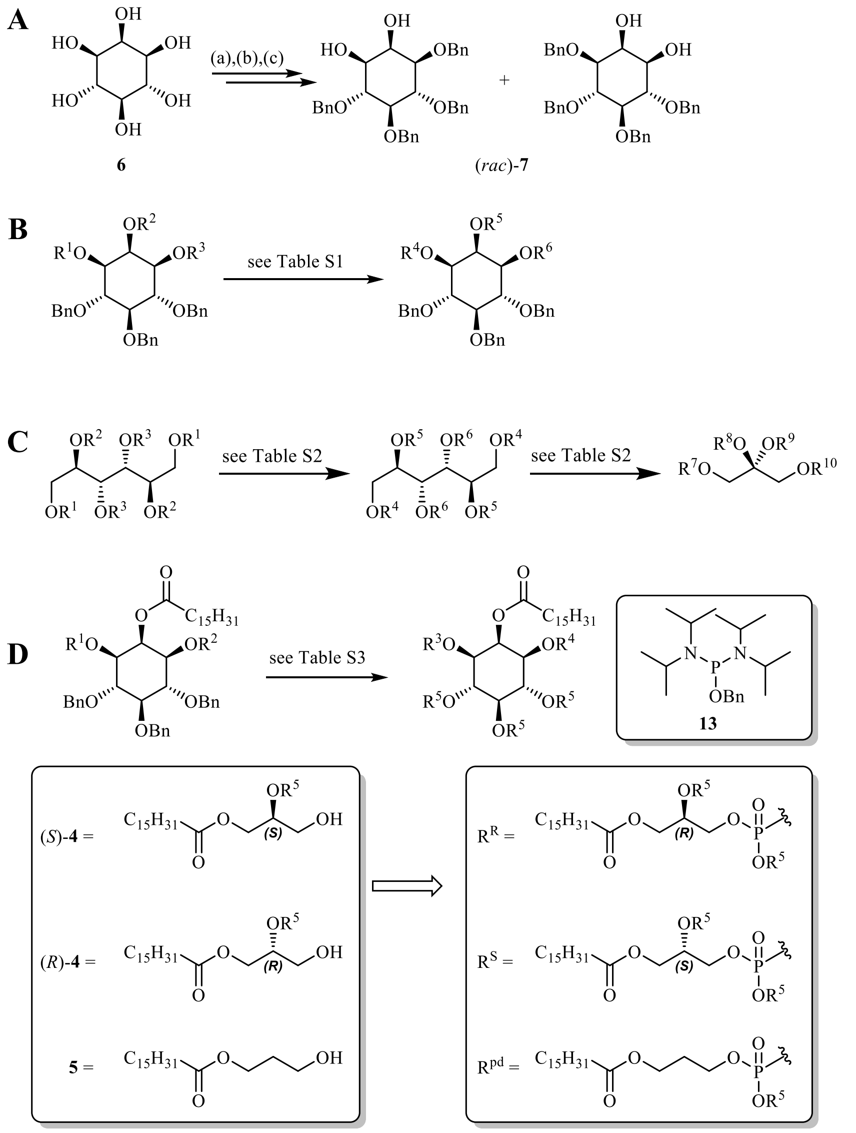

Suppl. Figure S1: Synthesis of synthetic *Eh*PIb-analogs.

A: Reaction scheme for the synthesis of (*rac*)-**7**(a): 2,2-DMP, DMSO, 48%; (b): NaH, BnBr, DMF; (c): 80% HOAc, 85% over 2 steps. **B**: Reaction scheme for the synthesis of **3**, **7**-**12** (see Table S1), **C**: reaction scheme for the synthesis of (***R***)**-4**, (***S***)**-4**, **15**-**21** (see Table S2). **D**: Coupling reactions for **1a-d**, **D-2**, **L-2**, **22a-d**, **D-24** and **L-24** (see Table S3).

Supplementary tables

Table S1: Reactions and residues of Figure S1, **B**. camph = 1*S*-(-)-camphanoyl-

#	STARTING MATERIAL	R^1	R^2	R^3	PRODUCT	R^4	R^5	R^6	YIELD
**1**	**7**, camph-Cl	H/Bn	H	Bn/H	(*mix*)-**9**	camph/Bn	H	Bn/camph	89%
**2**	(*mix*)-**9**	camph	H	Bn	**8a**	H	camph	Bn	30%
**3**	(*mix*)-**9**	Bn	H	camph	**8b**	Bn	camph	H	27%
**4**	**8a**	H	camph	Bn	**D-7**	H	H	Bn	92%
**5**	**8b**	Bn	camph	H	**L-7**	Bn	H	H	93%
**6**	**D-7**	H	H	Bn	**D-10**	allyl	H	Bn	92%
**7**	**L-7**	Bn	H	H	**L-10**	Bn	H	allyl	79%
**8**	**D-10**	allyl	H	Bn	**D-11**	allyl	$COC_{15}H_{31}$	Bn	79%
**9**	**L-10**	Bn	H	allyl	**L-11**	Bn	$COC_{15}H_{31}$	allyl	88%
**10**	**D-11**	allyl	$COC_{15}H_{31}$	Bn	**D-12**	H	$COC_{15}H_{31}$	Bn	68%
**11**	**L-11**	Bn	$COC_{15}H_{31}$	allyl	**L-12**	Bn	$COC_{15}H_{31}$	H	52%
**12**	**D-12, 13**	H	$COC_{15}H_{31}$	Bn	**L-3**	P(OBn)N(*i*Pr)$_2$	$COC_{15}H_{31}$	Bn	73%
**13**	**L-12, 13**	Bn	$COC_{15}H_{31}$	H	**D-3**	Bn	$COC_{15}H_{31}$	P(OBn)N(*i*Pr)$_2$	78%

Table S2: Reactions and residues related to Figure S1, **C**, Bzl: Benzylidene,

	STARTING MATERIAL			STARTING MATERIAL/ PRODUCT			PRODUCT				
**PROD.**	**R^1**	**R^2**	**R^3**	**R^4**	**R^5**	**R^6**	**R^7**	**R^8**	**R^9**	**R^{10}**	**YIELD**
**16**	H	H	H	Bzl	H	Bzl	-	-	-	-	30%
**17**	Bzl	H	Bzl	Bzl	Bn	Bzl	-	-	-	-	94%
**15**	Bzl	Bn	Bzl	H	Bn	H	-	-	-	-	85%
**18**	H	Bn	H	$C_{15}H_{31}$	Bn	H	-	-	-	-	78%
**(*S*)-4**	-	-	-	$C_{15}H_{31}$	Bn	H	$C_{15}H_{31}$	H	Bn	H	84%
**19**	H	Bn	H	TBDMS	Bn	H	-	-	-	-	70% o.
**20**	-	-	-	TBDMS	Bn	H	TBDMS	Bn	H	H	2 steps
**21**	-	-	-	-	-	-	TBDMS	Bn	H	$C_{15}H_{31}$	87%
**(*R*)-4**	-	-	-	-	-	-	H	Bn	H	$C_{15}H_{31}$	88%

Table S3: Reaction conditions related to Figure S1, **D**.

STARTING MATERIALS	R^1	R^2	PRODUCT	R^3	R^4	R^5	YIELD

**L-3**, (*S*)-**4**	P(OBn)N(*i*Pr)$_2$	Bn	**22a**	R^{R}	Bn	Bn	52 %
**L-3**, (*R*)-**4**	P(OBn)N(*i*Pr)$_2$	Bn	**22b**	R^{S}	Bn	Bn	69 %
**D-3**, (*S*)-**4**	Bn	P(OBn)N(*i*Pr)$_2$	**22c**	Bn	R^{R}	Bn	51 %
**D-3**, (*R*)-**4**	Bn	P(OBn)N(*i*Pr)$_2$	**22d**	Bn	R^{S}	Bn	85 %
**22a**	R^{R},R^{5}=Bn	Bn	**1a**	R^{R}	H	H	23 %
**22b**	R^{S},R^{5}=Bn	Bn	**1b**	R^{S}	H	H	64 %
**22c**	Bn	R^{R},R^{5}=Bn	**1c**	H	R^{R}	H	35 %
**22d**	Bn	R^{S},R^{5}=Bn	**1d**	H	R^{S}	H	31 %
**5, L-3**	P(OBn)N(iPr)$_2$	Bn	**L-24**	R^{pd}	Bn	Bn	67 %
**5, D-3**	Bn	P(OBn)N(*i*Pr)$_2$	**D-24**	Bn	R^{pd}	Bn	62 %
**L-24**	R^{pd},R^{5}=Bn	H	**L-2**	R^{pd}	H	H	84 %
**D-24**	H	R^{pd},R^{5}=Bn	**D-2**	H	R^{pd}	H	80 %

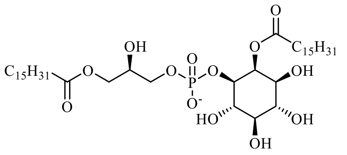

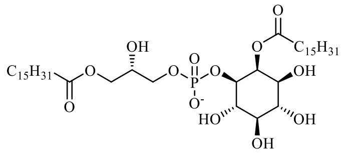

1a **1b**

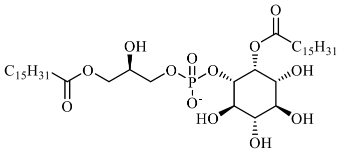

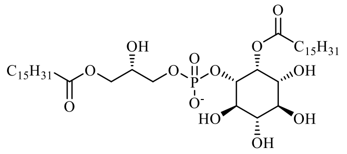

1c **1d**

1

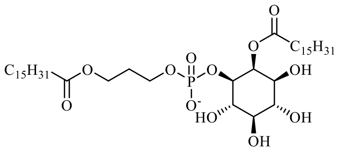

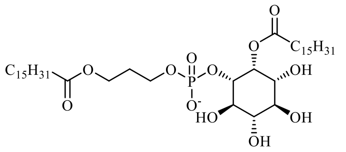

D-2 **L-2**

2

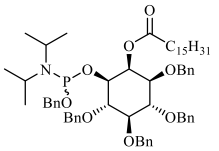

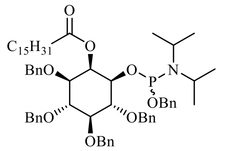

L-3 **D-3**

3

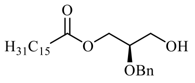

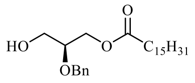

(*R*)-**4** (*S*)-**4**

4

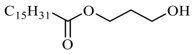

5

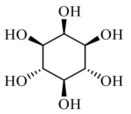

6

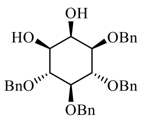

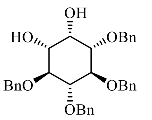

D-7 **L-7**

7

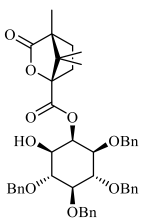

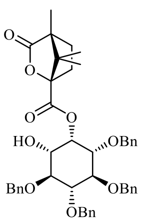

8a **8b**

8

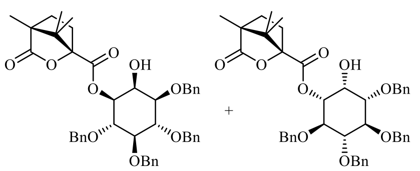

(*mix*)-**9**

9

10 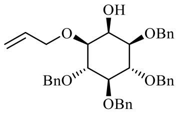 D-10 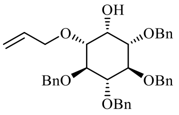 L-10

11 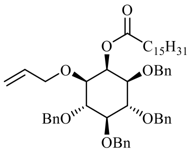 D-11 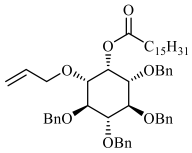 D-11

12 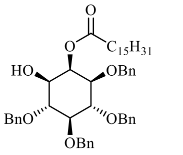 D-12 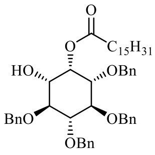 D-12

13 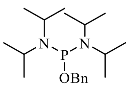

14 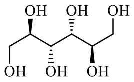

15 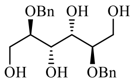

16 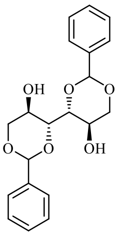

17 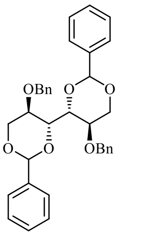

18 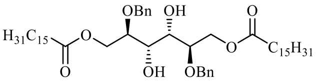

19 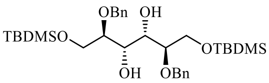

20 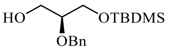

21 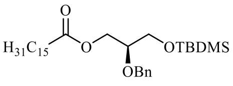

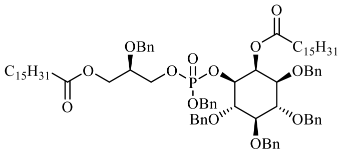
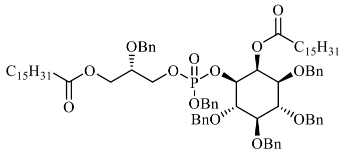

22a **22b**

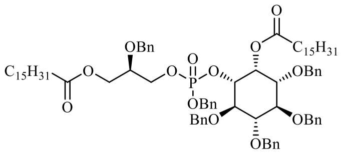
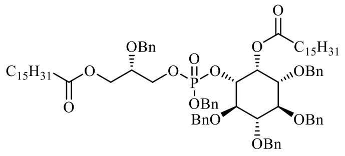

22 **22c** **22d**

23 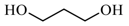

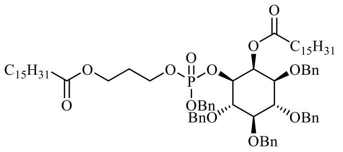
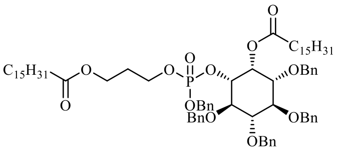

24 **D-24** **L-24**

Supplementary references

1. Gigg J, Gigg R, Payne S, Conant R. 1985. (±)-1,2:4,5-Di-O-isopropylidene-myo-inositol. Carbohydrate Research 142:132–134.
2. Cid M, Bonilla Julia B, Alfonso F, Martín-Lomas M. 2004. Synthesis of New Hexosaminyl D‐ and L‐chiro‐Inositols Related to Putative Insulin Mediators. European Journal of Organic Chemistry 2003:3505-3514.
3. Nkambule CM, Kwezi NW, Kinfe HH, Nokwequ MG, Gammon DW, Oscarson S, Karlsson E. 2011. Efficient regioselective protection of myo-inositol via facile protecting group migration. Tetrahedron 67:618-623.
4. Swarts BM, Guo Z. 2011. Chemical synthesis and functionalization of clickable glycosylphosphatidylinositol anchors. Chem Sci 2:2342-2352.
5. Alais J, Maranduba A, Veyrieres A. 1983. Regioselective mono-o-alkylation of disaccharide glycosides through their dibutylstannylene complexes. Tetrahedron Letters 24:2383-2386.
6. Smith AB, Rivero RA, Hale KJ, Vaccaro HA. 1991. Phyllanthoside-phyllanthostatin synthetic studies. 8. Total synthesis of (+)-phyllanthoside. Development of the Mitsunobu glycosyl ester protocol. Journal of the American Chemical Society 113:2092-2112.
